# Datasets depicting mobility retardation of NCS proteins observed upon incubation with calcium, but not with magnesium, barium or strontium

**DOI:** 10.1016/j.dib.2016.04.035

**Published:** 2016-04-21

**Authors:** Jeffrey Viviano, Anuradha Krishnan, Jenna Scully, Hao Wu, Venkat Venkataraman

**Affiliations:** a Graduate School of Biomedical Sciences, Rowan University, Stratford, NJ 08084, USA; b School of Osteopathic Medicine, Rowan University, Stratford, NJ 08084, USA

## Abstract

In this data article we show the specificity of the Ca^2+^-induced mobility shift in three proteins that belong to the neuronal calcium sensor (NCS) protein family: Hippocalcin, GCAP1 and GCAP2. These proteins did not display a shift in mobility in native gels when incubated with divalent cations other than Ca^2+^ – such as Mg^2+^, Ba^2+^, and Sr^2+^, even at 10× concentrations. The data is similar to that obtained with another NCS protein, neurocalcin delta (Viviano et al., 2016, “Electrophoretic Mobility Shift in Native Gels Indicates Calcium-dependent Structural Changes of Neuronal Calcium Sensor Proteins”, [1]).

**Specifications Table**

**Table:** 

Subject area	*Biology*
More specific subject area	*Electrophoretic Techniques*
Type of data	*Figure*
How data was acquired	*Electrophoresis: Bio-Rad miniPROTEAN*
Data format	*Analyzed*
Experimental factors	*For electrophoresis, standard protocols were used.*
Experimental features	*Divalent cations were tested in their ability to shift NCS proteins on Native gel*
Data source location	*Stratford, New Jersey, USA*
Data accessibility	*Data is within this article*

**Value of the data**

1. NCS proteins primarily bind and respond to calcium.
2. However, they have been shown to bind other metal ions, particularly Mg^2+^ [2].
3. Binding of Mg^2+^ was shown to affect the function of GCAP1 [3].
4. The data presented reveal that the mobility retardation on native gels is specifically induced only by calcium and not by other tested divalent cations – Mg^2+^, Ba^2+^, and Sr^2+^, even at 10× concentrations.
5. It may be possible to correlate binding of small ligands to structural changes detectable as electrophoretic mobility shift.

## 1 Data

**Fig. 1:**
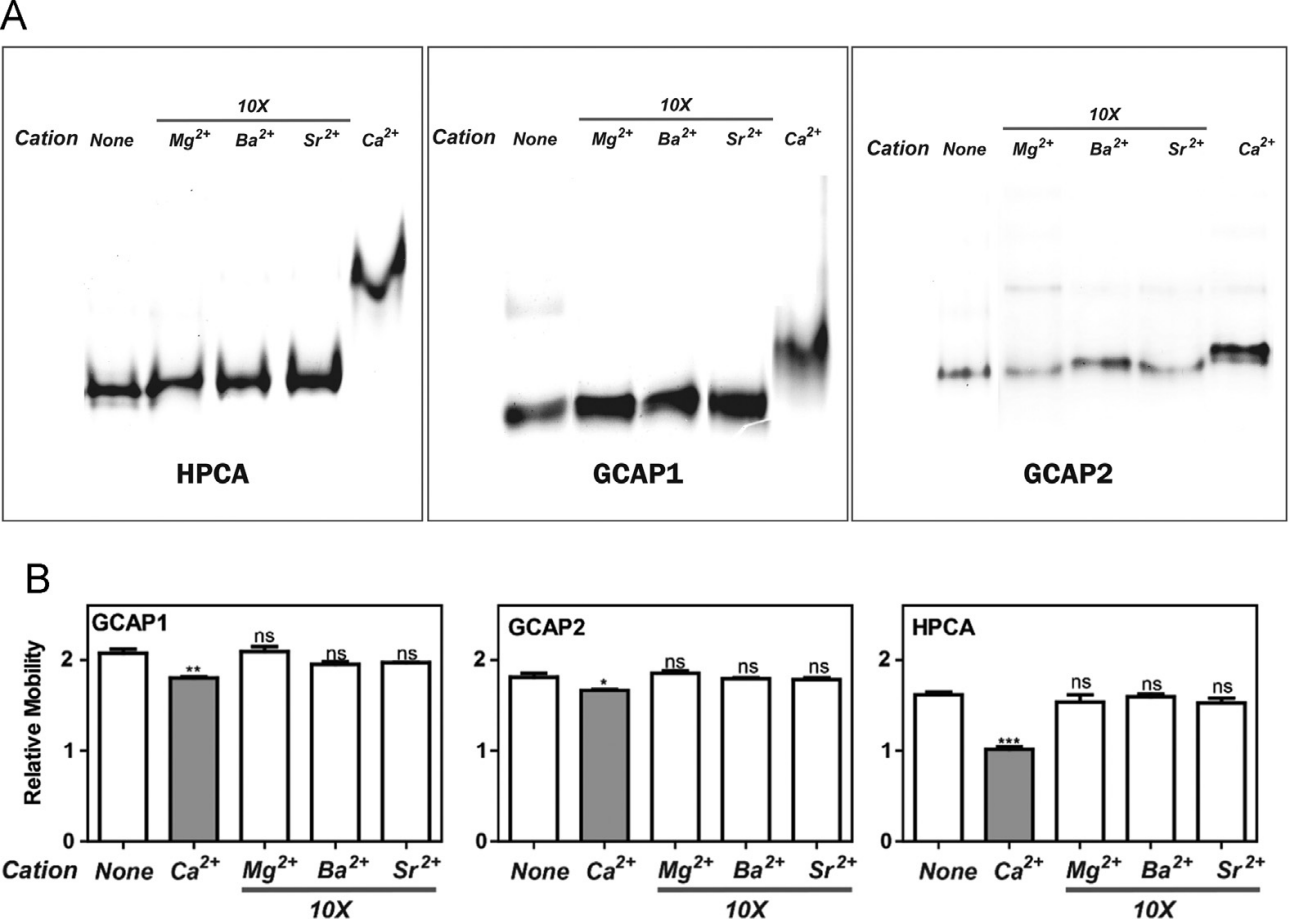
Effects of divalent cations on mobility of HPCA, GCAP1 and GCAP2 in native gels.

Bacterially expressed proteins – Hippocalcin (HPCA), Guanylate Cyclase Activating Proteins 1 and 2 (GCAP1 and GCAP2) – were purified in their myristoylated forms. The proteins were incubated with the divalent cation (Ca^2+^, Mg^2+^, Ba^2+^, or Sr^2+^) and were subjected to electrophoresis in native gels. Representative images are provided in Fig. 1A. Data was compiled from at least two independent preparations of the proteins with three independent replicates from each preparation. The mobility values were determined. The data (mean+SEM) is presented as a bar graph (Fig. 1B). Results from Student *t*-tests are also presented for each group against the control group (**, *P*<0.05; **, *P*<0.01; ***, *P*<0.001; ns – not significant). Mobility retardation is observed with the addition of calcium but not with any of the other divalent cations, even at concentrations ten times that of calcium.

## 2 Experimental design, materials and methods

In order to determine if divalent cations such as magnesium, strontium or barium could induce a mobility shift in the same way that calcium can [1], analyses were carried out with the proteins HPCA, GCAP1 and GCAP2. Proteins were expressed in *E. coli* ER2566 as described for NCALD in [1]. Briefly, cells grown overnight were inoculated (1% inoculum) into fresh LB medium and grown to an optical density of 0.6 at 600 nm. IPTG (1 mM final concentration) was then added for induction. For myristoylation, cells with yeast *N*-Myristoyl Transferase were used and myristic acid was supplemented. Cells were collected 2.5 h after induction, sonicated and the protein was purified. The purified protein was then washed with calcium-depleted Tris–Cl (20 mM; pH 7.5) to remove any residual calcium. Calcium removal was through the use of Chelex-100 resin (BioRad Laboratories, CA, USA) using standard procedures [4]. Proteins were then individually incubated in the presence of divalent cations (at indicated concentration within parentheses): calcium (39 µM) or magnesium (~400 µM), strontium (~400 µM) or barium (~400 µM). Electrophoreses in native gels and analyses were carried out as described [1].
